# DNA Methylation and Histone Modifications Are the Molecular Lock in Lentivirally Transduced Hematopoietic Progenitor Cells

**DOI:** 10.1155/2015/346134

**Published:** 2015-04-19

**Authors:** Siew Ching Ngai, Rozita Rosli, Akram Al Abbar, Syahril Abdullah

**Affiliations:** ^1^Medical Genetics Laboratory, Clinical Genetics Unit, Faculty of Medicine and Health Sciences, UPM, 43400 Serdang, Selangor, Malaysia; ^2^School of Biosciences, Faculty of Science, The University of Nottingham Malaysia, Jalan Broga, 43500 Semenyih, Selangor, Malaysia; ^3^Genetics & Regenerative Medicine Research Centre, Faculty of Medicine and Health Sciences, UPM, 43400 Serdang, Selangor, Malaysia

## Abstract

Stable introduction of a functional gene in hematopoietic progenitor cells (HPCs) has appeared to be an alternative approach to correct genetically linked blood diseases. However, it is still unclear whether lentiviral vector (LV) is subjected to gene silencing in HPCs. Here, we show that LV carrying green fluorescent protein (GFP) reporter gene driven by cytomegalovirus (CMV) promoter was subjected to transgene silencing after transduction into HPCs. This phenomenon was not due to the deletion of proviral copy number. Study using DNA demethylating agent and histone deacetylase (HDAC) inhibitor showed that the drugs could either prevent or reverse the silencing effect. Using sodium bisulfite sequencing and chromatin immunoprecipitation (ChIP) assay, we demonstrated that DNA methylation occurred soon after LV transduction. At the highest level of gene expression, CMV promoter was acetylated and was in a euchromatin state, while GFP reporter gene was acetylated but was strangely in a heterochromatin state. When the expression declined, CMV promoter underwent transition from acetylated and euchromatic state to a heterochromatic state, while the GFP reporter gene was in deacetylated and heterochromatic state. With these, we verify that DNA methylation and dynamic histone modifications lead to transgene silencing in HPCs transduced with LV.

## 1. Introduction

Gene therapy is the introduction of therapeutic genes into target cells to treat a disease or medical disorder [1]. Successful gene therapy requires specific, efficient, stable, and high levels of gene transfer into the target cells in order to achieve the therapeutic effects. Hematopoietic progenitor cells (HPCs) are attractive target cells for many blood diseases such as *β*-thalassemia and sickle cell disease as well as other hematological malignancies due to their ability to self-renew and reconstitute all lineages in the hematolymphoid system [2]. Lentiviral vectors (LVs) are promising tools in gene therapy for hematological diseases because they can efficiently transduce HPCs, which are quiescent and difficult to target [3].

However, several literatures have reported a decline in transgene expression in early progenitor cells and stem cells transduced with LVs over time in murine embryonic carcinoma P19 cells, human bladder carcinoma T24 cells, human breast carcinoma MDA-MB-231 cells, and others [4, 5]. Epigenetic effects were found as the confounding factor for the transgene expression decline in several cell types [4–7]. The epigenetic mechanisms, which have been proposed as the factors for transgene silencing, are DNA methylation, histone modifications, and microRNAs [8, 9]. Although studies on epigenetic effects on transgene expression have been performed on different cell types [4–7], none have been conducted on HPCs transduced by LV.

Therefore, in this study, we seek to (i) determine the gene expression profiles in HPCs transduced with LV and to (ii) examine whether DNA methylation and histone deacetylation are the factors for gene silencing in LV gene delivery. Identification and understanding of the factors for gene silencing could lead to the efforts in preventing the silencing effects in HPCs transduced with LVs. The extended expression of therapeutic gene in HPCs would lead to an efficient gene therapy to hereditary blood diseases with a single or periodic dosing.

## 2. Methodology

### 2.1. Isolation, Purification, Verification, and Enrichment of HPCs

Bone marrow cells were flushed from femur and tibia of BALB/c mice using recommended medium (phosphate buffered saline (PBS) + 2% fetal bovine serum (FBS) + 1 mM ethylenediaminetetraacetic acid) with a syringe equipped with a 23-gauge needle into a 100 mm diameter tissue culture plate. This step was repeated several times to flush all of the bone marrow cells out from the marrow cavities. Clumps were dispersed by gently passing the cell suspension through the syringe repeatedly. The cells were transferred into a 15 mL centrifuge tube. The cells were centrifuged at 250 ×g for 5 minutes at 4°C. Then, the supernatant was discarded and the cell pellet was resuspended in 1 mL of recommended medium with 2% normal rat serum. The amount of the cells was counted and then prepared at a concentration of 1 × 10^8^ cells/mL in a 5 mL polystyrene tube. The cells were subjected to Lin negative selection step, followed by cKit positive selection step by EasySep Magnetic Nanoparticles Separation (StemCell Technologies, Inc., Vancouver, BC, Canada) according to the manufacturer's instructions. The purified Lin^−^cKit^+^ cells were verified by flow cytometry analysis and the fold of cells enrichment was determined.

### 2.2. Duration of Transgene Expression

Viral transduction of purified HPCs was performed using Stemspan, SFM medium supplemented with 100 ng/mL recombinant human interleukin-11, 100 ng/mL human FMS-like tyrosine kinase 3 ligand, 50 ng/mL recombinant murine stem cell factor (Stemcells Technologies), and 40 *μ*g/mL lipoprotein low density from human plasma (Sigma Aldrich). HPCs were plated at 5 × 10^4^ cells/well in a 24-well plate. HPCs were transduced with LV carrying green fluorescent protein (GFP) reporter gene with the multiplicity of infection (MOI) of 3.2 (Optimal MOI, results not shown) in the presence of Polybrene (6 *μ*g/mL). LV-containing medium was discarded and replaced with fresh complete culture medium at day 1 after transduction. The GFP expression was measured at 6 hours and at days 1, 2, 3, 5, and 7 after transduction using flow cytometry. The results were evaluated as the percentage of GFP positive cells within total events acquired at different time points.

### 2.3. Relative Transgene Copy Number

LV-transduced HPCs and untreated HPCs were prepared following the protocol described above. The cells were harvested at day 2 and day 7 after transduction. DNA was extracted from 1 × 10^5^ cells using DNeasy Blood and Tissue kit (Qiagen). The concentration and the purity of DNA were determined by Nanodrop 2000 (Thermo Scientific, Wilmington, DE). The samples were subjected to polymerase chain reaction (PCR) with GoTaq DNA polymerase (Promega) in the provided GoTaq Flexi buffer under the following conditions: (i) for hypoxanthine-guanine phosphoribosyltransferase (HPRT) gene: 95°C for 2 minutes, followed by 35 cycles of 95°C for 1 minute, 56.6°C for 1 minute, and 72°C for 1 minute, with a final extension at 72°C for 5 minutes; (ii) for transgene: 95°C for 2 minutes, followed by 35 cycles of 95°C for 1 minute, 47°C for 1 minute, and 72°C for 1 minute, with a final extension at 72°C for 5 minutes. The PCR primers were 5′ HPRT gene, 5′-GCTGGTGAAAAGGACCTCT-3′; 3′ HPRT gene, 5′-CACAGGACTAGAACACCTGC-3′; 5′ transgene, 5′-ATAAGCTTGGGAGTTCCGCG-3′; 3′ transgene, 5′-AAAGCTGGGTTTACTTGTACAG-3′. The PCR products were subjected to agarose gel electrophoresis at 75 volts for 40 minutes. The intensity of the band was determined by Fluorchem FC2 system (Alpha Innotech Corporation, USA).

### 2.4. Cells Treatment with 5-Azacytidine and Trichostatin A

HPCs were transduced with LV as described above. To prevent the silencing, different concentrations of 5-azacytidine (5-azaC) (1, 2, 5, and 10 *μ*M) were added to the medium the following day of transduction. To reverse the silencing, different concentrations of 5-azaC (1, 2, 5, and 10 *μ*M) were added on the day of transgene silencing (day 3 after transduction). On the following day of 5-azaC treatment, 5-azaC-containing medium was discarded and replaced with fresh complete medium. At day 5 after transduction, the cells were harvested and centrifuged at 250 ×g for 5 minutes. The supernatant was discarded and the cell pellet was resuspended in PBS containing 1% FBS.

The experimental protocol was repeated for the study of Trichostatin A (TSA) at different concentrations (50, 100, and 150 nM) and the combination of 5-azaC and TSA at different concentrations (2 *μ*M 5-azaC + 50 nM TSA and 2 *μ*M 5-azaC + 150 nM TSA). The cells were dissociated and centrifuged at 250 ×g for 5 minutes to remove residual media components. Next, the cell pellet was resuspended in PBS containing 1% FBS. Total event of 10,000 was set. The cells were analyzed for green fluorescence by FACSvantage (Becton Dickinson, San Jose, CA) using CellQuest software. Dead cells were excluded and negative green fluorescence was set at around 1% for the untreated cells.

### 2.5. Sodium Bisulfite Genome Sequencing

The transduced cells were harvested at day 2 and day 7 after transduction. DNA was extracted from 1 × 10^5^ cells using DNeasy Blood and Tissue kit (Qiagen) following the manufacturer's instructions. The concentration and the purity of DNA were determined by Nanodrop 2000 (Thermo Scientific, Wilmington, DE).

Methylated pL/CMV/GFP (1 *μ*g) (served as the positive control) and the genomic DNA (1 *μ*g) from the transduced cells harvested at day 2 and day 7 after transduction were subjected to restriction enzyme digestion prior to bisulfite conversion. All the samples were digested with 10 U of* EcoR1* and* AvaI* in 1x NE buffer 4 in a total reaction volume of 50 *μ*L. The reaction mixture was incubated at 37°C for 1 hour. The restriction digested DNA was purified from the enzymatic reaction by using MinElute Reaction Cleanup Kit (Qiagen). The DNA fragments between 70 bp and 4 kb were purified. The purified DNA was chemically treated with sodium bisulfite mix (85 *μ*L) and then purified by using EpiTect Bisulfite Kit (Qiagen). The purified DNA from bisulfite conversion reaction was subjected to PCR with GoTaq DNA polymerase (Promega) in the provided GoTaq Flexi buffer under the following conditions: 95°C for 2 minutes, followed by 35 cycles of 95°C for 1 minute, 52.9°C for 1 minute, and 72°C for 1 minute, with a final extension at 72°C for 5 minutes. The PCR primers targeting CMV promoter region were as follows: 5′ CMV promoter, 5′-ATAAGCTTGGGAGTTCCGCG-3′; 3′ CMV promoter, 5′-CCTCTAGAGTCGGTGTCTTCT-3′. PCR product was verified by agarose gel electrophoresis. The bands were visualized by Fluorchem FC2 system (Alpha Innotech Corporation, USA). The fresh verified PCR products were cloned into pCR 2.1-TOPO by TOPO TA Cloning Kit (Invitrogen) and transformed into JM109 competent cells on the same day. The inoculum was subjected to DNA purification using PureLink HQ Mini Plasmid Purification Kit. The purified DNA was subjected to sequencing by using pCR 2.1-TOPO M13 reverse primer.

### 2.6. Chromatin Immunoprecipitation Assay

The transduced HPCs were harvested at day 2 and day 7 after transduction. The cells were subjected to ChIP assay by using ChIP Kit (Abcam, Inc., Cambridge, MA). The chromatins were labelled with (i) anti-histone H3 acetyl K9 (anti-H3K9) antibody (ab10812; Abcam), (ii) anti-histone H3 dimethyl lysine 4 (anti-H3 diMeK4) antibody (ab32356; Abcam), (iii) anti-histone H3 dimethyl lysine 9 (anti-H3 diMeK9) antibody (ab1220; Abcam), and (iv) negative control (Beads only), respectively. Anti-H3K9 antibody was used to detect H3 acetylation; anti-H3 diMeK4 antibody is a marker for euchromatin; anti-H3 diMeK9 antibody is a marker for heterochromatin. The samples were incubated overnight with rotation at 4°C. To pull down antibody-DNA-protein, 50 *μ*L of DNA purifying slurry was added to the samples. The samples were mixed by inversion and incubated at 98°C for 10 minutes. After incubation, the samples were left at room temperature for 20 minutes to cool. Proteinase K (1 *μ*L) was added to the samples and then vortexed for 5 seconds at medium power. The samples were incubated at 30 minutes at 55°C and then 10 minutes at 98°C. DNA slurry was pelleted by centrifugation at 14000 ×g for 1 minute at room temperature. The supernatant was transferred to a clean 1.5 mL tube. PCR-grade H_2_O (65 *μ*L) was added to the DNA slurry and then vortexed for 10 seconds at medium power. DNA slurry was pelleted by centrifugation at 14000 ×g for 1 minute at 4°C and the supernatant was transferred to a clean 1.5 mL tube. The purified DNA after ChIP assay was subjected to PCR with GoTaq DNA polymerase (Promega) in the provided GoTaq Flexi buffer under the following conditions: (i) *β*-actin promoter: 95°C for 2 minutes, followed by 35 cycles of 95°C for 1 minute, 54.7°C for 1 minute, and 72°C for 1 minute, with a final extension at 72°C for 5 minutes; (ii) CMV promoter: 95°C for 2 minutes, followed by 35 cycles of 95°C for 1 minute, 52.9°C for 1 minute, and 72°C for 1 minute, with a final extension at 72°C for 5 minutes; (iii) GFP gene: 95°C for 2 minutes, followed by 35 cycles of 95°C for 1 minute, 47°C for 1 minute, and 72°C for 1 minute, with a final extension at 72°C for 5 minutes. The PCR primers used were 5′ *β*-actin promoter, 5′-ATGCTGCACTGTGCGGCGAG-3′; 3′ *β*-actin promoter, 5′-TGGCTGCAAAGAGTCTACACG-3′; 5′ CMV promoter, 5′-ATAAGCTTGGGAGTTCCGCG-3′; 3′ CMV promoter, 5′-CCTCTAGAGTCGGTGTCTTCT-3′; 5′ GFP gene, 5′-CTCCACCATGGTGAGCAA-3′; 3′ GFP gene, 5′-AAAGCTGGGTTTACTTGTACAG-3′. The PCR products were verified by agarose gel electrophoresis at 75 volts for 40 minutes. The bands were visualized by Fluorchem FC2 system.

### 2.7. Statistical Analysis

Numerical data was expressed as mean with standard deviations. ANOVA was applied to compare the means of the experiment samples, followed by* Post hoc* test to determine the statistical significance in the mean difference. Differences of *P* < 0.050 were considered statistically significant.

## 3. Results and Discussion

### 3.1. Isolation, Purification, Verification, and Enrichment of HPCs

The hallmark of HSCs is their lifelong reconstitution of the multilineage hematopoiesis potential in the transplanted hosts. Identification and isolation of such cells have challenged researchers for decades [10]. Up to date, the primitive HSCs of adult murine bone marrow are isolated by Lin^−^Sca^+^cKit^+^ selection [11]. However, Sca-1 expression on HSCs is variable in different mouse strains [12]. The expression of Sca-1 in BALB/c mouse is lower compared to C57BL/6J mouse. The use of Sca-1 in HSCs isolation from BALB/c mice will reduce the amount of the purified HSCs because many of the primitive HSCs, which do not express Sca-1, will be lost during the isolation step. Therefore, we selected Lin^−^cKit^+^ instead of using the Lin^−^Sca^+^cKit^+^ for the cells isolation from BALB/c mouse and the isolated cells are termed HPCs.

**Figure 1:**
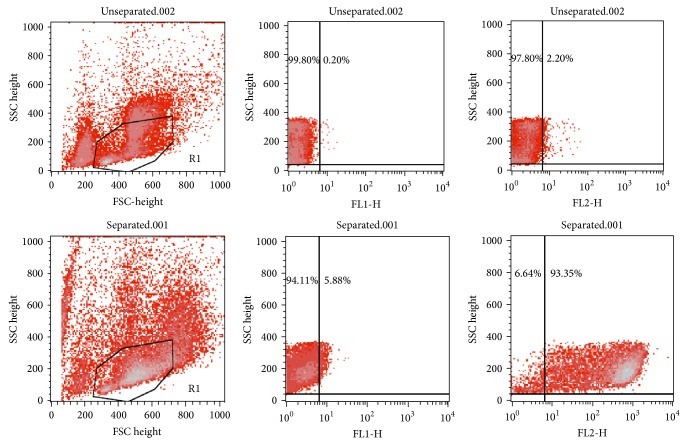
The verification of the isolated Lin^−^cKit^+^ cells by flow cytometry analysis. Before HPC purification, the percentage of HPCs was 2.20. After purification, the percentage of HPCs was 93.35.

Before the isolation by using EasySep Magnetic Cell Separation, the percentage of Lin^−^cKit^+^ cells was 2.20%, verified by flow cytometry analysis. After the isolation steps, the percentage of Lin^−^cKit^+^ cells was 93.35%. The results of flow cytometry analysis are shown in Figure 1. The enrichment of HPCs was 42-fold. The HPCs isolated were highly enriched. Therefore, this separation method had successfully depleted most of the unwanted cells and enriched the murine HPCs prior to experimental steps.

### 3.2. Duration of the Transgene Expression in HPCs

**Figure 2:**
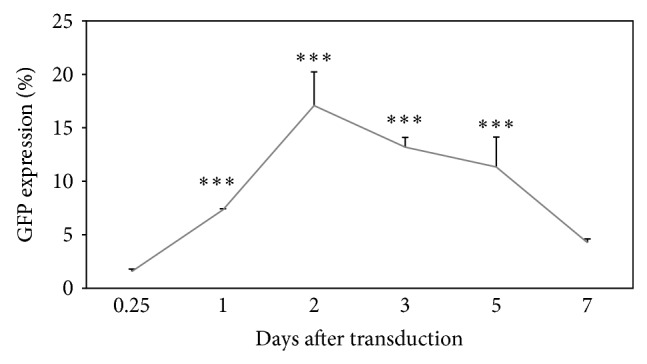
GFP expression of HPCs transduced with LV/CMV/GFP at MOI of 3.2 at different time points. The GFP expression was measured, at days 0.25 (6 hours), 1, 2, 3, 5, and 7 after transduction as the percentage of GFP-expressing cells within the total events acquired. Data are presented as mean ± standard deviation in triplicate. Statistical difference between groups compared to the untreated group is reported as ^∗∗∗^ *P* < 0.001.

The transduction efficiency and duration of transgene expression of HPCs transduced with LV were examined* in vitro*. The GFP expression was determined at 6 hours and at days 1, 2, 3, 5, and 7 after transduction.  Figure 2 shows rapid transgene silencing in HPCs transduced with the LV. The GFP expression increased from 1.57% at 6 hours to 7.34% at day 1 after transduction. The highest level of transgene expression was read at day 2 after transduction (17.08%) (*P* < 0.001). The GFP expression decreased starting from day 2 after transduction, reaching values of 4.07% at day 7 after transduction (*P* < 0.001). LV transgene silencing was also observed in many cell types although the rate of silencing varies depending on the cell types [4, 13, 14]. Since the GFP expression started to decrease at day 2 after transduction, therefore, day 3 was chosen as the day of transgene silencing for the following experiments.

### 3.3. Relative Transgene Copy Number

To exclude the contribution of the loss of the integrated provirus to the transient gene expression, the genomic DNA was extracted from the untransduced cells and transduced cells at day 2 and day 7 after transduction. The extracted genomic DNA was subjected to PCR with the primers specific to the proviral transgene and with the primers specific to HPRT gene as a control.

**Figure 3:**
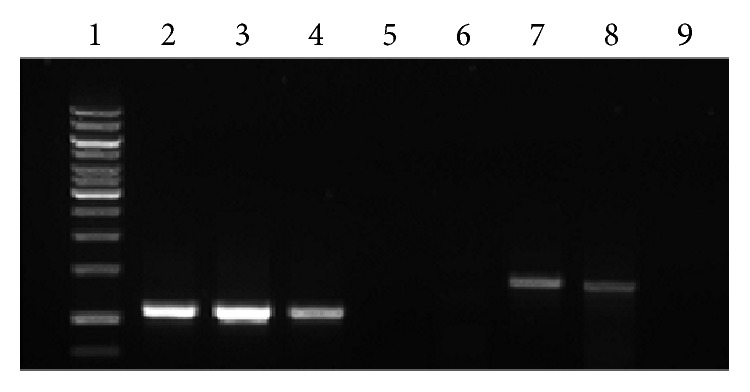
Relative transgene copy number. The extracted genomic DNA from the transduced cells at day 2 and 7 after transduction was subjected to PCR to amplify the transgene region of the sample. Lane 1: 1 kb ladder; Lanes 2–4: PCR with primers specific for HPRT gene; Lane 2: Untransduced genomic DNA (positive control for PCR); Lane 3: transduced genomic DNA extracted at day 2 after transduction (positive control for relative transgene copy number); Lane 4: transduced genomic DNA extracted at day 7 after transduction (positive control for relative transgene copy number); Lane 5: PCR reaction without DNA (negative control); Lane 6–9: PCR with primers specific to transgene; Lane 6: untransduced genomic DNA; Lane 7: transduced genomic DNA extracted at day 2 after transduction; Lane 8: transduced genomic DNA extracted at day 7 after transduction; Lane 9: PCR reaction without DNA (negative control for PCR).

Figure 3 shows that the expected bands were obtained in Lanes 2, 3, and 4 which were set as the controls, indicating that the mouse housekeeping HPRT gene was expressed in both untransduced and transduced murine HPCs harvested at day 2 and day 7 after transduction. No band was obtained in Lane 5, which was set as negative control for PCR. For PCR with primers specific to the transgene, no band was obtained for the untransduced cells (Lane 6), indicating no integrated provirus in the cells. Expected bands were obtained for the LV-transduced cells harvested at day 2 and day 7 after transduction, indicating that the provirus was integrated into the cells genome. No band was obtained for PCR reaction mixture without DNA, which was set as negative control.

The relative transgene copy number was determined by dividing the band intensity of HPRT gene with band intensity of transgene at day 2 and day 7 after transduction, respectively. The gene copy number of HPRT gene was set as 2 and the relative transgene copy numbers for the transduced cells were 0.62 at day 2 and 0.92 at day 7 after transduction. This shows that the relative provirus DNA copy number remained almost the same. Therefore, we believe that the transgene silencing was caused by certain silencing machineries and not due to the loss of proviral DNA. This is consistent with the results shown by He et al. [4], in which the decrease of transgene expression by LV at the very early stage after gene transfer was due to transcriptional silencing in murine embryonic carcinoma P19 cells and not due to the deletion of the transgene. Besides that, our results were further supported by other research groups which reported that the primary mechanism that limits the long-term episomal expression is caused by gene silencing, rather than loss of vector DNA [15, 16]. Following this, we further investigated the factors of transgene silencing in the following experiments.

### 3.4. DNA Methylation as the Factor for Transgene Silencing

**Figure 4:**
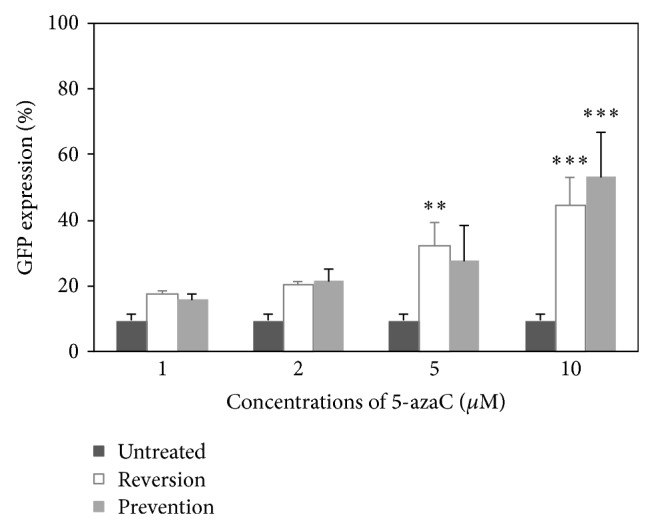
GFP expression of HPCs transduced with LV/CMV/GFP and treated with 1, 2, 5, and 10 *μ*M 5-azaC to prevent and to reverse GFP silencing. The expression was measured at day 5 after transduction. Untreated cells were served as the control. Data are presented as mean ± standard deviation in triplicate. Statistical difference between groups compared to the transduced untreated group is reported as ^∗∗^ *P* < 0.010 and ^∗∗∗^ *P* < 0.001.

As 5-azaC is a demethylating agent, its effect on the prevention of transgene silencing and reversion of a silenced gene may implicate DNA methylation as the cause for the short-lived transgene expression. HPCs were transduced with LV and the LV-containing medium was discarded at day 1 after transduction. 5-azaC was added on the day after the LV transduction to prevent the transgene silencing (prevention study) or on the day of transgene silencing to reverse the silenced gene (reversion study). Based on the results obtained shown in Figure 4, 5-azaC was able to prevent and reverse the silencing effect in a dose-dependent manner. In the reversion study, the GFP expression was 17.37%, 20.52%, 32.22%, and 44.48% for the cells treated with 1, 2, 5, and 10 *μ*M 5-azaC. The GFP expression was 15.70%, 21.50%, 27.70%, and 53.31%, respectively, for 1, 2, 5, and 10 *μ*M of 5-azaC added to prevent the silencing effects. The cells treated with 5-azaC at different concentrations for both prevention and reversion studies showed a trend of higher GFP expression when compared to the transduced 5-azaC-untreated cells (9.27%). However, not all the values were statistically significant.

Based on the results obtained, 5-azaC could prevent and reverse DNA methylation in a dose-dependent manner. Our results on the prevention study corroborates the study by Escher et al. [17], which showed that 5-azaC prevented the methylation of the CMV promoter in the transfected murine macrophage RAW 264.7 cells. Besides that, Di Ianni et al. [18] reported that the 5-azaC prevented transgene methylation in severe combined immunodeficiency disease mice injected with herpes simplex virus type 1 thymidine kinase gene/bacterial beta-galactosidase gene- (*LacZ*-) transduced human monocytic U937 cells. 5-azaC was found to integrate into the DNA and prevent methylation by inhibiting* Dnmt* [19]. For the reversion study, our results substantiate the study by Ou et al. [5] who reported that methylated genes could be restored by 5-azaC. In addition, He et al. [4] also demonstrated that 5-azaC was able to reactivate the* LacZ* expression in a dose-dependent manner in murine embryonic carcinoma P19 cells transduced with LV. These are in agreement with Lu and Richardson [20] who reported that 5-azaC could covalently bind to* Dnmt* and thus deplete cellular* Dnmt* pools, resulting in the hypomethylation of the nascent DNA strand.

### 3.5. Histone Deacetylation as the Factor for Transgene Silencing

Besides DNA methylation, histone acetylation and deacetylation have been shown to determine the transcriptional activity of the chromatin [21]. HDAC and other corepressor proteins are recruited by MBD or MECP2 to the methylated region of DNA to repress the transcription in a methylation-dependent manner [22]. The effect of HDAC inhibitor, TSA, on the prevention of transgene silencing and reversion of a silenced gene was analyzed to examine whether histone deacetylation was associated with the short-lived transgene expression. TSA with different concentrations (50, 100, and 150 nM) was added a day after transduction to prevent GFP silencing or on the day of transgene silencing to reverse the silenced GFP. The GFP expression was checked at day 5 after transduction.

**Figure 5:**
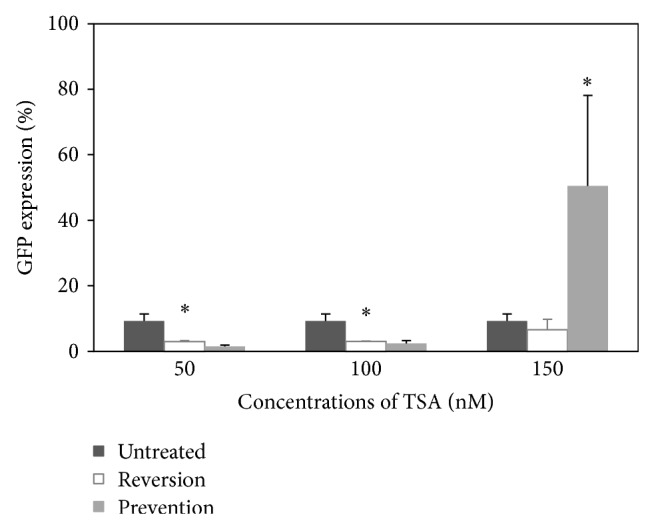
GFP expression of HPCs transduced with LV/CMV/GFP and treated with 50, 100, and 150 nM TSA to prevent and to reverse GFP silencing. The expression was measured at day 5 after transduction. Untreated cells were served as the control. Data are presented as mean ± standard deviation in triplicate. Statistical difference between groups compared to the transduced untreated group is reported as ^∗^ *P* < 0.050.

The GFP expression of the transduced cells without TSA treatment was 9.27% (Figure 5). For the reversion of transgene silencing study, the GFP expression was 2.91%, 2.99%, and 6.57% for the cells treated with 50, 100, and 150 nM TSA, respectively. The GFP expression of the transduced cells treated with 50 and 100 nM TSA was significantly lower compared to the transduced untreated cells (*P* = 0.015 and *P* = 0.017, resp.). There was no significant difference in GFP expression between the transduced untreated cells with the transduced cells treated with 150 nM TSA (*P* = 0.373). The result shows that TSA was unable to reverse the silenced GFP. This may be because histone deacetylation process has already occurred on the day of transgene silencing. Therefore, the addition of TSA has no effect, as it could only act on the activity of HDAC.

For the prevention of transgene silencing study, the GFP expression was 1.48% and 2.37% for the cells treated with 50 and 100 nM TSA, respectively, which were lower than the control transduced untreated cells. The GFP expression of the cells treated with 150 nM TSA (50.43%) was significantly higher than the transduced untreated cells and the transduced cells treated with 50 and 100 nM TSA (*P* < 0.050). It is speculated that the treatment of TSA before gene silencing could inhibit HDAC's activity and consequently generated higher GFP expression compared to the untreated cells. Since the histone deacetylation process is a balance of HDAC and HAT (histone acetyltransferase) activity, a decrease in TSA concentration can reduce histone acetylation and affects the transgene expression [5]. Therefore, there was no effect on GFP expression for the cells treated with lower concentrations of TSA (50 nM and 100 nM).

### 3.6. DNA Methylation and Histone Deacetylation as the Factors for Transgene Silencing

It was reported that the reexpression of the silenced gene could be achieved in the presence of DNA demethylating agents and HDAC inhibitors [23]. Ou et al. [5] also reported that the drugs that affect the chromatin modifications would not affect the expression of methylated gene unless if they were used in combination with inhibitors of* Dmnt*. Furthermore, Ma et al. [24] reported that blockade of histone deacetylation by TSA prevents DNA methylation by decreasing its effect on* Dnmt1* expression.

**Figure 6:**
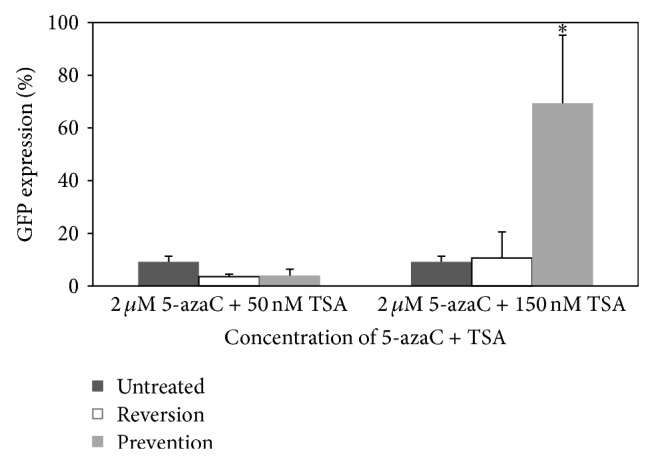
GFP expression of HPCs transduced with LV/CMV/GFP and treated either with 2 *μ*M 5-azaC + 50 nM TSA (Cocktail 1) or with 2 *μ*M 5-azaC + 150 nM TSA (Cocktail 2) to prevent and to reverse GFP silencing. The expression was measured at day 5 after transduction. Untreated cells were served as the control. Data are presented as mean ± standard deviation in triplicate. Statistical difference between groups compared to the transduced untreated group is reported as ^∗^ *P* < 0.050.

Therefore, the effect of the combination of both drugs in preventing and reversing the transgene silencing was investigated after analyzing their individual effects. 5-azaC and TSA combinations (Cocktail 1: 2 *μ*M 5-azaC + 50 nM TSA; Cocktail 2: 2 *μ*M 5-azaC + 150 nM TSA) were added a day after transduction to prevent GFP silencing or on the day of GFP silencing to reverse the silenced GFP. The GFP expression in the reversion study was 3.56% and 10.67% for Cocktail 1 and Cocktail 2, respectively (Figure 6). Unfortunately, the readings were not statistically different from the transduced untreated cells (9.27%). The GFP expression was low for the cells treated with Cocktail 1 in the prevention study. However, significantly high expression (70.00%) was achieved for the transduced cells treated with Cocktail 2 (*P* = 0.006).

Based on the results obtained, our results further confirmed that the combination of 5-azaC and TSA could synergistically influence the GFP expression when the cells were treated before the silencing occurred. On the reversion attempt of the silenced gene treated with the drug cocktails, our results demonstrated that when the HDACs and corepressors have already bound to the methylated DNA, the addition of the drug cocktails could not reverse the deacetylation process.

### 3.7. Sodium Bisulfite Sequencing

Partial reactivation of transgene expression by 5-azaC suggests that DNA methylation plays some role in transgene silencing in murine embryonic carcinoma P19 cells [4]. Based on the results obtained, 5-azaC was able to prevent and reverse the silencing effect. Therefore, we further examined the methylation status in CMV promoter region by sodium bisulfite sequencing. The unmethylated cytosine was converted to uracil after bisulfite treatment and read as thymine by sequencing, while the methylated cytosine residues remained unchanged.

As a positive control, CMV promoter of pL/CMV/GFP was methylated by CpG methylase* in vitro* (positive controls 1, 2, and 3). Based on the sequencing results obtained, CMV promoter region was methylated by CpG methylase successfully (Table 1). The CpG methylation efficiency by the methylase was up to 85.2% (methylated cytosine in CpG/totalCpG sites × 100%). The other cytosine residues, which were not CpG motifs, were chemically converted to uracil by the bisulfite treatment. The efficiency of bisulfite conversion was 93.5% (amount of converted cytosine/amount of unmethylated cytosine × 100%).

**Figure 7:**
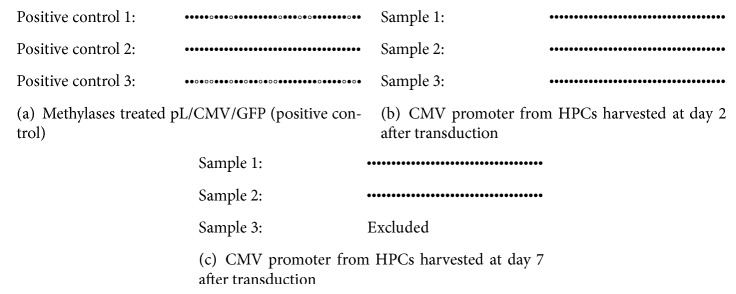
Bisulfite genomic sequencing of CpG in the methylated CMV promoter region. (a) Methylase treated pL/CMV/GFP (positive control), (b) CMV promoter from HPCs harvested at day of gene expression (day 2 after transduction), and (c) CMV promoter from HPCs harvested at day of gene silencing (day 7 after transduction). One circle corresponds to one cytosine in CpG. The total number of cytosine in the CMV promoter is 36. The filled circle corresponds to methylated CpG and the open circle corresponds to unmethylated CpG.

For the samples harvested at day 2 and day 7 after transduction, all the cytosine residues in the CpG motifs of the CMV promoter remained as cytosine, which means that all cytosine residues in the CpG sites were methylated (Figure 7). The result from day 7 sample 3 (third-triplicate for sample harvested at day 7 after transduction) was excluded as mutation occurred. There was no difference in the CMV promoter methylation pattern from the transduced cells harvested at day 2 and day 7 after transduction. The cytosine residues in the promoter region were found to be methylated as early as day 2 after transduction, during the peak of GFP expression. The methylation status was maintained until day 7 when the GFP expression declined. He et al. [4] reported that the packaging binding site plus packaging signal (PBS + *ψ*) region was heavily methylated when the transgene expression was high. In contrast, it was reported that the CpG methylation in the EF1*α* promoter progresses gradually in accordance to the reduction in transgene expression. In this study, we used the EpiTect Bisulfite Kit (Qiagen) for bisulfite conversion, which is reputed to convert unmethylated cytosine to more than 90% efficiency. This claim has been substantiated by the result from Table 1. In addition, the sequencing was performed in triplicate. These measures should improve the fidelity of the results.

Other literatures have shown that DNA methylation of promoter/enhancer region in oncoretroviral vectors was implicated in transgene silencing [25, 26]. These results suggest that transgene silencing is initiated by DNA methylation at the promoter region. However, distinct methylation kinetics patterns were observed for the primer binding site and packaging signal regions, which were the CpG-rich regions. These CpG-rich regions were hypermethylated as early as day 5 after transduction, even before the transgene has been silenced [4]. This report compliments our result, which shows that CpG in CMV promoter was methylated during the period of high transgene expression. Nevertheless, this confounding result will need further investigation.

### 3.8. Chromatin Immunoprecipitation Assay

Histone methylation can lead to transcriptionally active or inactive state of chromatin, depending on the amino acid residues being methylated [27]. Methylation of lysine 4 at the H3 tail has been associated with transcriptional active gene whereas methylation of lysine 9 at the H3 tail has been associated with transcription silencing [28–30]. Methylation of lysine 4 at H3 tail is a marker for euchromatin whereas methylation of lysine 9 at H3 tail is a typical code for heterochromatin in eukaryotic cells [4].

To further expand the analysis, the chromatin modifications in the CMV promoter and GFP reporter gene were examined. An open chromatin structure is often associated with histone H3 acetylation and active transcription. To examine whether the GFP silencing in HPCs transduced with LV was caused by the chromatin modifications, ChIP assay was performed on both the nonsilenced (day 2 after transduction) and the silenced (day 7 after transduction) HPCs. To determine whether there was histone code difference before and after transgene silencing, anti-H3 diMeK4 antibody and anti-H3 diMeK9 antibody were used in our study.

**Figure 8:**
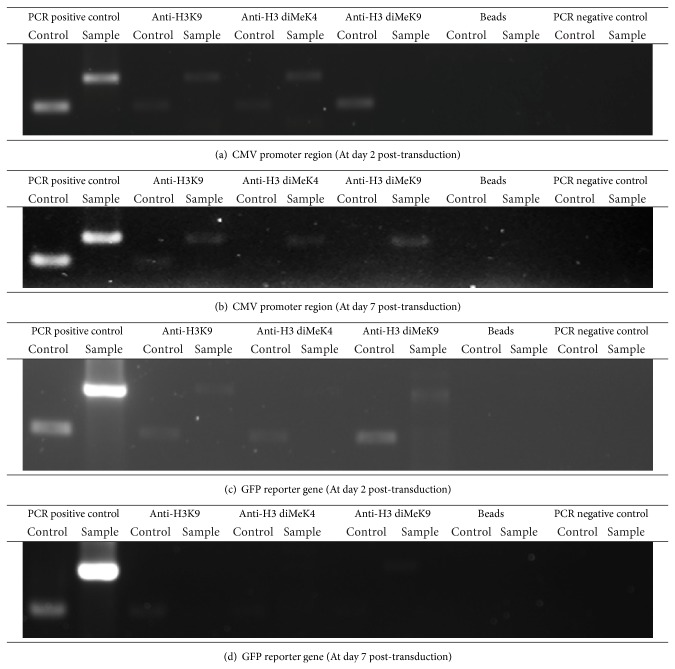
ChIP assay of CMV promoter region and GFP reporter gene at day 2 and day 7 post-transduction. The chromatin of the non-silenced and silenced HPCs were harvested at day 2 and day 7 post-transduction and immunoprecipitated with anti-histone H3 acetyl lysine 9 (Anti-H3K9) antibody, anti-histone H3 dimethyl lysine 4 (Anti-H3 diMeK4) antibody and anti-histone H3 dimethyl lysine 9 (Anti-H3 diMeK9) antibody. The DNA was subjected to PCR with primers specific to either CMV promoter region or GFP reporter gene and *β*-actin promoter region (As positive control for ChIP assay). The chromatin harvested without immunoprecipitation (Beads) was set as a negative control for the ChIP assay. The PCR reaction mixture without DNA was set as the negative control for PCR.

As a housekeeping gene, *β*-actin has an open chromatin structure under all conditions. Therefore, the *β*-actin promoter was used as a positive control in the ChIP assay. Based on the results obtained, *β*-actin promoter was enriched by anti-H3K9 antibody in both nonsilenced (Figures 8(a) and 8(c)) and silenced (Figures 8(b) and 8(d)) transduced HPCs. In CMV promoter region, anti-H3K9 antibody was also enriched in both nonsilenced (Figure 8(a)) and silenced (Figure 8(b)) transduced HPCs. This suggests that the local H3 was in acetylated status before and after transgene silencing. CMV promoter region of the nonsilenced cells was enriched with anti-H3 diMeK4 antibody but not with anti-H3 diMeK9 antibody (Figure 8(a)), a finding consistent with the euchromatic pattern in transcriptionally active region. However, for the silenced cells harvested at day 7 after transduction, both anti-H3 diMeK4 antibody and anti-H3 diMeK9 antibody were enriched in CMV promoter region (Figure 8(b)).

CMV promoter was enriched with both anti-H3K9 and anti-H3 diMeK4 antibodies at day 2 after transduction. Therefore, the histones in the region of the CMV promoter were in a euchromatic state at day 2 after transduction. This is consistent with the finding by He et al. [4]. For the murine embryonic carcinoma P19 cells transduced with the LV, the EF1*α* promoter was found to be in a euchromatin state in the positively expressed cells [4]. For the CMV promoter region extracted from HPCs harvested at day 7 after transduction (silenced cells), the histones were in a transition from euchromatin to heterochromatic state, since both anti-H3 diMeK4 and anti-H3 diMeK9 antibodies were enriched.

The results obtained from the sodium bisulfite sequencing indicate that the CMV promoter was methylated at day 2 after transduction. However, the results obtained from ChIP assay indicates that the acetylation of histone still remained at similar day. He et al. [4] reported that the CpG methylation might not correlate with histone modifications because the packaging signal region in transcriptionally active state was quickly hypermethylated regardless of hyperacetylation status. Furthermore, euchromatin state of histones was still detected in our study. Therefore, we speculate that the CMV promoter region was in a transitional period from acetylated (euchromatin state) before being completely deacetylated (heterochromatin state).

After the chromatin status has been determined in the CMV promoter region in both nonsilenced and silenced cells, we examined the chromatin modifications in GFP reporter gene in the cells harvested at day 2 and day 7 after transduction. As shown in Figure 8(c), anti-H3K9 antibody was enriched in GFP gene of nonsilenced cells, which is consistent with the transcriptionally active state. This process could reduce the positive charge and weaken the interaction of histone with DNA, consequently, increasing the transcriptional activity. In contrast, no anti-H3K9 antibody was enriched in GFP reporter gene for the cells harvested at day 7 after transduction, in which the GFP expression declined (Figure 8(d)). The deacetylated histones could increase the positive charge of the histone and strengthen the interaction between histone and DNA. As the result, the entry and the binding of transcription factors to the region were inhibited.

Strangely, at day 2 after transduction, anti-H3 diMeK9 antibody was enriched in GFP gene but not anti-H3 diMeK4 (Figure 8(c)). This shows that although the GFP expression was high at day 2 after transduction, the GFP gene was in a heterochromatic state. Therefore, we speculate that although the region surrounding the GFP reporter gene is condensed, the GFP will still be expressed as long as the CMV promoter which controls the GFP expression is acetylated and is in a euchromatin state. At day 7 after transduction, anti-H3 diMeK9 was also enriched in GFP gene. This shows that GFP gene was in a heterochromatic state which is in agreement with a transcriptionally inactive period (Figure 8(d)). Overall, these results indicate that GFP reporter gene was in heterochromatic state at day 2 and day 7 after transduction even though GFP expression was detected at day 2 after transduction.

Table 2 summarizes our discoveries on the methylation and chromatin status of the CMV promoter and GFP transgene during the peak and lowest transgene expression from LV-transduced HPCs. The CMV promoter was found to be methylated at the very early stage of transgene expression, similar to the status at day 7 after transduction. As for the chromatin status, the CMV promoter was acetylated and was in euchromatic state at day of highest transgene expression and was acetylated and was in heterochromatic state at day of transgene silencing. Strangely, the GFP transgene was acetylated but was in heterochromatic state at day of highest transgene expression and was deacetylated and was in heterochromatic state at day of transgene silencing. These results suggest that the change in histone modifications in the promoter region correlates well with the initiation of transgene silencing. Our results support the study by He et al. [4] that transgene silencing is dominated by the status of the promoter.

## 4. Conclusion

Our study suggests that DNA methylation and dynamic histone modifications are involved in the transgene silencing of HPCs transduced by LV. The lentiviral expression vector without CpG is expected to be free from methylation; therefore, it is expected that there will be no recruitment of transcriptional repressor complexes to silent the gene expression. Boundary elements or insulators can also be inserted in the lentiviral construct to prevent the encroachment of heterochromatin into the promoter and/or transgene region. It is hoped that with these proposed strategies, the transgene silencing problems in HPCs transduced with LV could be solved and long-term therapeutic gene expression could be achieved.

## Figures and Tables

**Table 1:** Percentage efficiency of CpG methylase and bisulfite conversion for positive control of sodium bisulfite sequencing.

Reaction	Efficiency (%)			Average (%)
	Positive Control 1	Positive Control 2	Positive Control 3	
Efficiency of CpG methylase	83.3	100	72.2	85.2
Efficiency of bisulfite conversion	92.8	93.3	94.4	93.5

**(a):** 

Day 2 Highest GFP Expression	CMV Promoter	GFP Transgene
Chromatin Status	Acetylated/Euchromatic	Acetylated/Heterochromatic
Methylation status	Methylated	

**(b):** 

Day 7 Lowest GFP Expression	CMV Promoter	GFP Transgene

Chromatin Status	Acetylated/Heterochromatic	Deacetylated/Heterochromatic
Methylation status	Methylated	
